# Switch from Olanzapine Long-Acting Injectable to its Oral Equivalent during COVID-19 Pandemic: a Real World Observational Study

**DOI:** 10.1007/s11126-021-09924-9

**Published:** 2022-03-02

**Authors:** Ana-Aliana Miron, Andreea Teodorescu, Petru Ifteni, Claudia Alexandrina Irimie, Lorena Dima, Paula-Simina Petric

**Affiliations:** Transilvania University of Brasov, 56 Nicolae Bălcescu Str., 500019 Brasov, Romania

**Keywords:** Schizophrenia, Olanzapine pamoate, Long-acting antipsychotic, Relapse

## Abstract

Schizophrenia is a psychiatric condition with chronic evolution, one of the most disabling diseases. The main cause for the disease’s progression is considered to be the lack of compliance with the treatment. Long-acting injectable antipsychotics (LAIs) are an important treatment option for patients with schizophrenia. Olanzapine long-acting injection (OLZ-LAI) is a pamoate monohydrate salt of olanzapine that is administered by deep intramuscular gluteal injection. The aim of this paper is to report the effects of a sudden and unplanned switch from olanzapine long-acting injectable to oral olanzapine in remitted patients with schizophrenia due to restrictions caused by the COVID-19 pandemic. An observational study conducted in the Clinical Hospital of Psychiatry and Neurology of Brasov, Romania between April 2020 and March 2021. 27 patients with OLZ-LAI were entered into the study. Of 27 cases, 21 patients preferred to be switched to oral olanzapine (77.77%). Only 6 patients continued with the long-acting formulation. The main reason for the initiation of olanzapine pamoate in all the patients was non-adherence to oral medication (80.95%), and the mean age of starting LAI olanzapine was 36.42 years (SD ± 10.09). Within the following 12 months after switching from olanzapine LAI to OA, 15 patients (71.42%) relapsed, and 12 were admitted to the emergency psychiatric unit. The COVID-19 pandemic has brought multiple disservices to current medical practice. Sudden and unplanned switch from olanzapine long-acting formulation to oral olanzapine was followed by the high rate of relapse in remitted schizophrenia.

## Introduction

Schizophrenia is a psychiatric condition with a chronic evolution and a disabling disorder. One of the well-established cause for the disease’s progression is antipsychotic nonadherence.

Long-acting injectable antipsychotics (LAIs) are an important treatment option for patients with schizophrenia who have difficulties in adhering to oral regimens. Long-acting formulations ensure that the patient has received the treatment. Since regulations require that the injections are administered in healthcare facilities, it is immediately known when the patient does not return for treatment [1, 2].

The paradigm for LAIs has changed from the early 2000s, when LAIs were recommended for non- adherent patients or for patients with multiple relapses. The new guidelines recommend these formulations as the treatment of choice as soon as possible after the onset of schizophrenia, in order to avoid relapses and brain damages [3, 4].

Olanzapine long-acting injection (LAI) is a pamoate monohydrate salt of olanzapine that is administered by deep intramuscular gluteal injection. The efficacy and tolerability profile of olanzapine LAI is similar to that of oral olanzapine [5, 6]. The “post-injection delirium/sedation syndrome” (PDSS) was the “scariest” side effect despite its low incidence (approximately 0.07% of injections) [7]. The major reason for non-acceptance of olanzapine pamoate treatment was the protocol of administration. The recommendation was that the injection had to be administered by qualified personnel in a medical setting. After the administration, the patients had to remain under observation for 3 h and then they could only leave the hospital if accompanied by another person [8]. These measures generated higher costs for the hospitals (treatment room arrangement, monitoring room, etc.) but were balanced by the better cost-effectiveness ratio of LAI compared with OA [9].

However, patients on LAI treatment had the longest period of remission of the disease and some of the best adherences to treatment [10]. Its efficacy has been reported even in preventing relapses in the catatonic form of schizophrenia [11].

Despite its proven effectiveness, high cost, stigma, and fear determined a world-wide underuse of LAIs [12]. The patient’s refusal of injectable treatment is related to the administration mode, the treatment control, the administration protocols (in hospitals or in specialized healthcare centers, post-injection monitoring in the case of olanzapine pamoate, etc.) [13].

To all of the above, the COVID-19 pandemic added new restrictions or limitations in prescribing and administration of LAIs [14, 15]. The aim of this paper was to present the outcomes after an abrupt and unplanned switch from olanzapine long-acting injection (OLZ-LAI) to oral olanzapine (O-OLZ). The Hospital Ethics’ Committee entitled “Comisia de Etică” approved this research.

## Methods

27 patients treated with OLZ-LAI were recorded in March 2020 in the documents of the Clinical Hospital of Psychiatry and Neurology of Brasov, Romania, an academic emergency psychiatric unit with 3 departments and 150 beds. In order to limit the possibility of contamination, after the State of Emergency was declared in Romania (March 15, 2020), the authorities have recommended limited or restricted access to hospitals, which remain strictly intended for emergencies alone.

As a result, the public compartment for the administration of LAIs (including olanzapine pamoate) was closed. Given that, schizophrenia patients treated with olanzapine LAI were advised to continue the administration in another setting (e.g. private clinics). Due to high costs (around 30 euro/injection), working schedule in the private practices, the long distance from home, and anxiety generated by the new situation, 21 schizophrenia patients preferred to be switched from OLZ-LAI to oral olanzapine. Only 6 patients continued the treatment with the long-acting formulation. They agreed to participate in this observational study and provided signed informed consent.

21 patients started oral olanzapine treatment 30 days after the last OLZ-LAI administration according to the following model:

- 2 × 210 mg injections/month were replaced with 15 mg oral olanzapine/day
- 1 × 300 mg injection/month was replaced with 10 mg oral olanzapine/day
- 2 × 300 mg injections/month were replaced with 20 mg oral olanzapine /day
- 1 × 405 mg injection/month was replaced with 15 mg oral olanzapine/day

We compared the patients’ outcomes in both groups: ***Switched from OLZ-LAI*** **vs.** ***Stay on OLZ-LAI.***

### Statistical Analysis

Demographic, clinical and olanzapine-related characteristics of patients treated with OLZ-LAI and O- OLZ were calculated using basic statistic. Variables scores before and after OLZ-LAI exposure were compared using the variance ratio test (F-test). Statistical significance was set at two-sided *p* < 0.05.

## Results

The results showed that those who switched from LAI to the oral form of olanzapine relapsed more frequently (15 out of 21 vs. 1 out of 6). For a clearer image of the two groups, we also presented also the data of the 27 subjects before the COVID-19 period. The demographics and clinical characteristics of patients switched to oral olanzapine are presented in Table 1.

**Table 1:** Demographics

Characteristic	Switched from OLZ-LAI *n* = 21	Stay on OLZ-LAI *n* = 6	*P* value
Age (years, mean, SD)	42.52 ± 10.15	49.5 ± 11.29	0.1591
Male gender (n, %)	9 (42.85%)	2 (33.3%)	0.6813
Smoking (n, %)	11 (52.38%)	3 (50%)	0.9455
Age of onset (years, mean, SD)	24.52 ± 4.45	23.45 ± 4.66	0.6114
Duration of illness (years, mean, SD)	18 ± 9.70	19.3 ± 11.1	0.7811
Duration of illness before olanzapine LAI (years, mean, SD)	11.42 ± 8.26	9.06 ± 7.99	0.5401
Reason for olanzapine LAI initiation			
Non-adherence (n, %)	17 (80.95%)	5 (83.3%)	0.9063
Side-effects to other antipsychotics	3 (14.28%)	1 (16.7%)	0.9452
Switch from clozapine	1 (4.77%)	–	–
Age at olanzapine LAI started (years, mean, SD)	36.42 ± 10.09	33.56 ± 9.2	0.5390
Duration of olanzapine LAI (years, mean, SD)	6.09 ± 1.51	5.56 ± 1.88	0.5291
Olanzapine LAI doses			
210 mg every 2 weeks (n, %)	2 (9.52%)	–	–
300 mg every 4 weeks (n, %)	11 (52.38%)	3 (50%)	0.9112
300 mg every 2 weeks (n, %)	6 (28.58%)	1 (16.7%)	0.8183
405 mg every 4 weeks (n, %)	2 (9.52%)	2 (33.3%)	0.6156
Number of episodes before olanzapine LAI initiation (years mean ± SD)	7.38 ± 2.15	8.41 ± 2.04	0.7734
Number of episodes after olanzapine LAI initiation (years mean ± SD)	0.57 ± 0.67	0.53 ± 0.74	0.1209

The main reason for the initiation of olanzapine pamoate was non-adherence to oral medication (80.95%), which is similar to the findings of previous studies [16]. The mean age of starting LAI olanzapine was 36.42 years (SD ± 10.09), similar to the mean age of patients in de Haya et al. study [17].

The longest treatment with olanzapine LAI was 9 years and the mean duration of olanzapine LAI treatment was 6.09 years (SD ± 1.51). There was a significant difference in the number of episodes before and after olanzapine LAI initiation (5.38 vs. 0.57, *p* < 0.0001). The most used dosage was 300 mg every 2 weeks and the most unchanged dose in time was 300 mg every 4 weeks. During the treatment period, the LAI group received more than 1000 injections with only 7 mild intensity PDSS (sedation, confusion, slurred speech), all fully recovered, and all but one patient continued the treatment after the event.

**Fig. 1:**
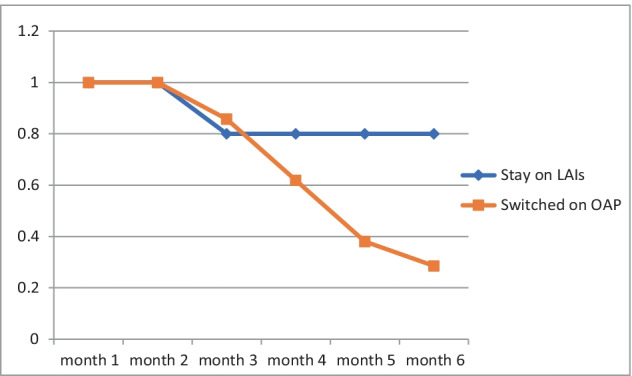
Kaplan-Meier survival analysis

The outcomes of the patients that were switched from OLZ-LAI to oral olanzapine were not favorable (Fig. 1). In the next 6 months, 15 patients (71.4%) relapsed, 12 (66.7%) needing hospitalization. Of these, 2 patients resumed treatment with OLZ-LAI, 3 were switched to another type of LAI (2 on aripiprazole and 1 on paliperidone). There was no statistically significant difference in the doses administered to those who relapsed compared to the 6 who did not relapse (361.1 mg ± 102.4 vs. 417.5 mg ± 134.9, *p* = 0.275).

## Discussion

To our knowledge, this is the first naturalistic study regarding the switch from olanzapine long-acting to oral olanzapine in patients with schizophrenia in clinical remission. Given the long period of remission before the COVID-19 pandemic, some of the patients were considered recovered (remission of symptoms, good level of functioning). They managed to have stable life partners and worked. Some even have children to take care of. The biggest problem in the pandemic was the OLZ-LAI 3-h surveillance model. During the COVID-19 pandemic this was not possible and clinicians refused to risk and to indicate administration at home, or with a shorter interval of monitoring (as the majority of reactions have been reported within the first hour after the injection) [18].

The switch to oral treatment, initially considered temporary (no one knew in March 2020 how long the pandemic will last), has shown that patients can become non-adherent again in an active (no longer want treatment) or passive (forget to take treatment) manner. This event led to relapse in over 70% of the patients. On the other hand, 5 out of 6 patients (83.33%) who continued with LAI remained in remission.

Olanzapine LAI provides a slow continuous release of olanzapine that continues for approximately six to eight months after the last injection (https://oxfordhealthbrc.nihr.ac.uk/our-work/oxppl/table-4-lai/). According to the experts’ recommendation we started with oral olanzapine when the next injection is due, with careful monitoring especially in the first two months after the discontinuation of olanzapine LAI. The patients started to relapse as seen in Fig. 1.

As far as we know, such studies are not published. In practice, LAIs are stopped for the main following reasons: i) switching from a first-generation LAI to a second-generation LAI; ii) patients no longer want to continue; iii) there is no evidence of efficacy [19].

The study’s limitation is the small sample size. The strength of the report lies in the patient’s characteristics (remission and long period of olanzapine LAI treatment) and the long follow-up period.

## Conclusions

The COVID-19 pandemic has brought multiple disservices to the current medical practice. One of these was the visible decrease in the initiations of OLZ-LAI and even the cessation of administration due to the restrictions imposed by the pandemic. Under these circumstances, even patients with long periods of remission relapsed after switching to oral treatment. It would seem that the previous duration of remission is not a protective factor when the patient becomes non-compliant with treatment again.

## Data Availability

The data that support the findings of this study are available from the corresponding author upon reasonable request. with the authorization of the hospital Ethics Committee.

## Abbreviations

LAI: Long-acting injectable antipsychotics
OLZ-LAI: Olanzapine long-acting injection
COVID-19: Coronavirus Disease-19
OA: oral antipsychotic
PDSS: post-injection delirium/sedation syndrome
O-OLZ: oral olanzapine
SD: standard deviation
